# Supplementary material for: Preventing erosion of X-chromosome inactivation in human embryonic stem cells
Source: Nat Commun. 2022 May 6;13:2516. doi: 10.1038/s41467-022-30259-x (PMC9076865; doi:10.1038/s41467-022-30259-x)
Supplement: Supplementary file 1 — Supplementary Information [file 41467_2022_30259_MOESM1_ESM.pdf]

Supplementary Table 1. hESC and Feeder Cell Sources and Identifiers

Cell Line	Source	Identifier
Inactive Human Foreskin Fibroblasts (HFFs)	Global Stem	Global Stem: #GSC-3002
Mouse Embryonic Fibroblasts (MEFs)	Global Stem	Global Stem: #GSC-6001G
UM33-4 hESC line (NIH approval number NIH hESC-14-0279)	MStem Cell Laboratories	hESC Line: UM33-4
UM63-1 hESC line (NIH approval number NIH hESC-14-0277)	MStem Cell Laboratories	hESC Line: UM63-1
UM77-2 hESC line (NIH approval number NIH hESC-14-0278)	MStem Cell Laboratories	hESC Line: UM77-2
UM90-14 hESC line (NIH approval number NIH hESC-15-0306)	MStem Cell Laboratories	hESC Line: UM90-14

Supplementary Table 2. Quantitative Real-Time PCR Primers

Gene	Forward Primer	Reverse Primer
OCT3/4	GATGGCGTACTGTGGGCCC	TGGGACTCCTCCGGGTTTTG
NANOG	TCCTCCTCTTCCTCTATACTAAC	CCCACAAATCACAGGCATAG
SOX2	GAGAGAAAGAAAGGGAGAGAAG	GAGAGAGGCAAACTGGAATC
β-ACTIN	GCCGAGGACTTTGATTGC	GTGTGGACTTGGGAGAGG
AFP	AAACTATTGGCCTGTGGCGA	GGCCAACACCAGGGTTTACT
GATA-4	CAGATGCCTTTACACGCTGA	TCCGCTTGTTCTCAGATCCT
BRACHY	ACCCAGTTCATAGCGGTGAC	GGATTGGGAGTACCCAGGTT
VE-CAD	CCTACCAGCCCAAAGTGTGT	GAGATGACCACGGGTAGGAA
TUJ-1	ATGCGGGAGATCGTGCACAT	CCCTGAGCGGACACTGT
KRT-18	CACAGTCTGCTGAGGTTGGA	GAGCTGCTCCATCTGTAGGG

Supplementary Table 3. hESC Line Karyotyping Results

Cell Line	Passage	Result	Notes
UM77-2	19	46,XX	Karyotyped
UM33-4	31	46,XX	Karyotyped
UM33-4	23	46,XX	24-Chromosome Molecular/Microarray PGS
UM63-1	22	46,XX	24-Chromosome Molecular/Microarray PGS
UM63-1	8	46,XX	Karyotyped; 18 cells with normal karyotype, 2 cells with non-clonal aberrations
UM63-1	20	46,XX	Karyotyped; 11 cells with normal karyotype, 7 cells had 3 X-chromosomes, 2 cells with non-clonal aberrations
UM90-14	6	46,XX	Karyotyped; 19 cells with a normal karyotype, 1 cell with a non-clonal chromosomal aberration

a

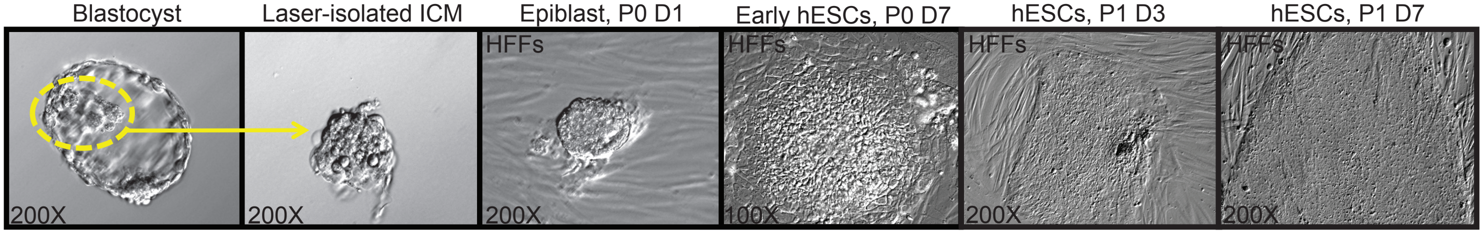

b

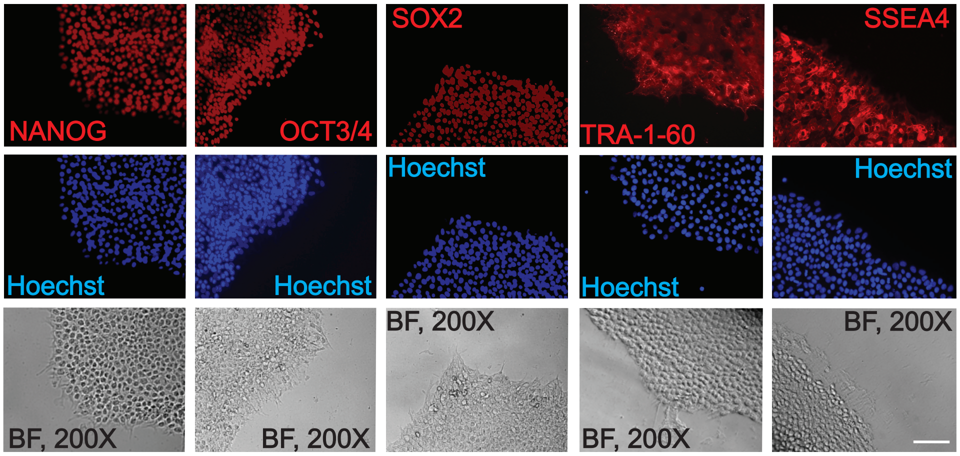

c

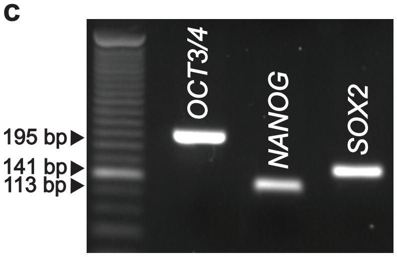

qPCR for hESC Pluripotency Markers	
**Transcript**	**CT***
NANOG	6.98
OCT3/4	5.25
SOX2	7.16
*CT calculated in relation to *ActB*	

d

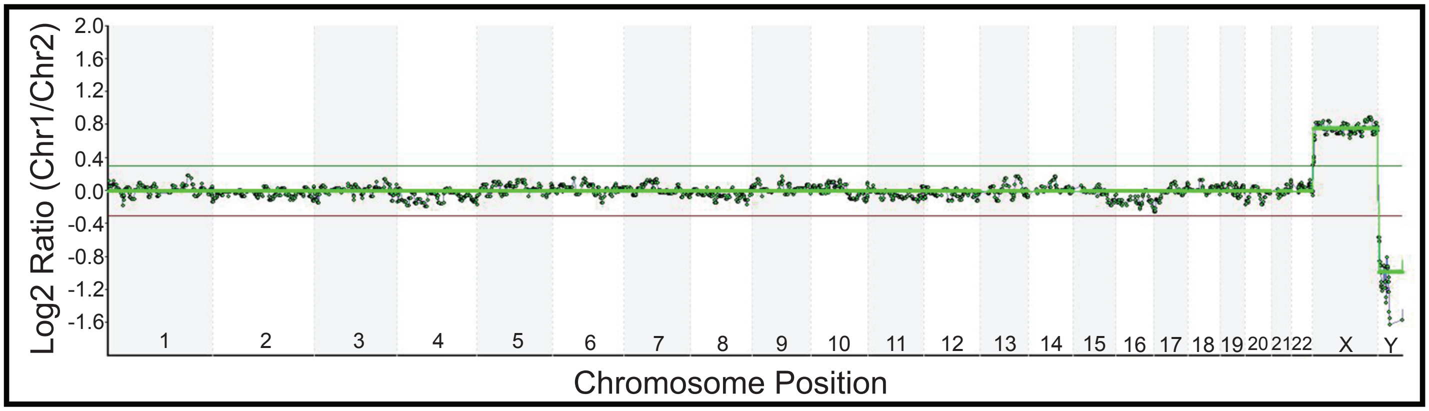

e

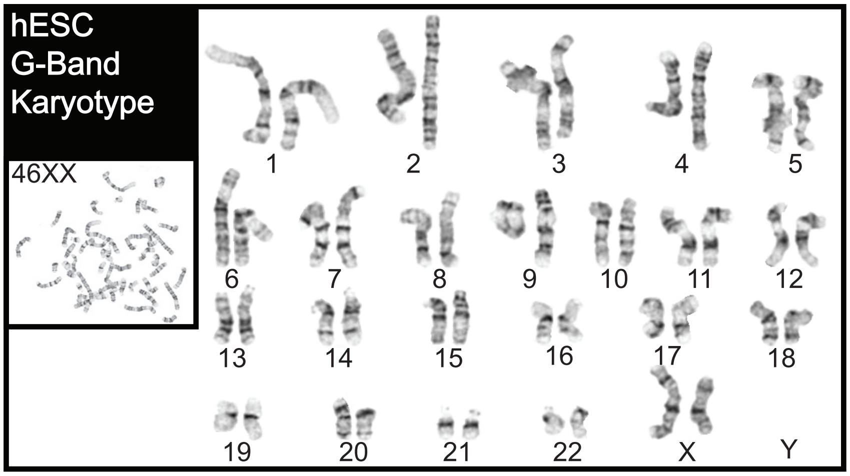

f

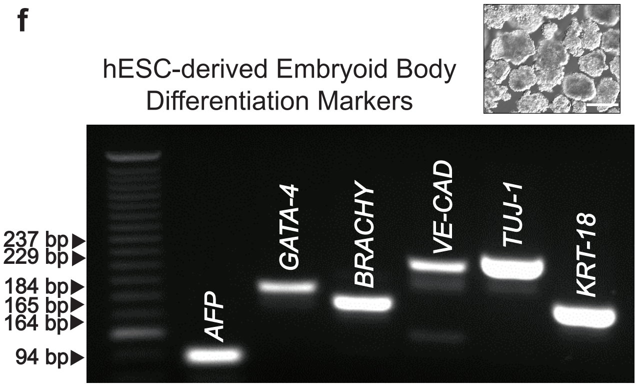

qPCR for D8 hESC-derived EB Differentiation Markers	
**Transcript**	**CT***
AFP (endoderm)	15.26
GATA-4 (endoderm)	17.38
BRACHY (mesoderm)	14.07
VE-CAD (mesoderm)	17.52
TUJ-1 (ectoderm)	6.56
KRT-18 (ectoderm)	5.23

Supplementary Fig. 1. Derivation and Characterization of hESCs. (a) Representative micrographs of day (D) 5 human blastocyst embryos following thawing; laser dissection of inner cell mass (ICM); plating of the ICM on human foreskin fibroblast feeder cells (HFFs) resulting in epiblast outgrowth; and, the resultant derivation of hESC colonies. Four blastocysts were used to generate four independent hESC lines for this study; micrographs were generated for each embryo and hESC line. **(b)** Representative micrographs of hESCs stained by immunofluorescence to detect pluripotency markers NANOG, OCT3/4, SOX2, TRA-1-60, and SSEA4 (red) with Hoechst nuclear staining (blue), and brightfield (BF) images to assess morphology. All four independent hESC lines generated for this study were subjected to immunofluorescent staining. Scale bar ~50 microns. **(c)** Top, RT-qPCR detection of the pluripotency markers *OCT3/4*, *NANOG*, and *SOX2*. Bottom, relative quantitation by RT-qPCR of *NANOG*, *OCT3/4*, and *SOX2* in relation to *β-ACTIN* (*ACTB*). RT-qPCR was conducted in triplicate for every transcript in each hESC line. Collectively these data (**b**, **c** top and bottom) demonstrate reproducibility of protein/transcript assays showing hESC line pluripotency. **(d)** Representative low passage number 24-chromosome molecular/microarray demonstrating normal karyotype (46,XX) of a female hESC line. **(e)** Representative G-band karyotype of metaphase spreads of a female hESC line. **(f)** Representative micrograph of hESC-derived embryoid bodies (EB, Day 8, inset, scale bar ~100 microns) and RT-qPCR detection of lineage markers (*AFT*, *GATA-4* – two independent endoderm markers; *BRACHY*, *VE-CAD* – two independent mesoderm markers; and *TUJ-1*, KRT-18 – two independent ectoderm markers). Bottom, relative quantitation by RT-qPCR of lineage markers in hESC-derived EBs normalized to *ACTB*. Collectively these data (**f**, top and bottom) demonstrate reproducibility of the

transcription assays of the ability of hESC line-derived EBs to form endoderm, mesoderm, and ectoderm. RT-qPCR was conducted in triplicate for each transcript for every hESC line. Each hESC line employed in this study was subjected to these analyses and only the female hESC lines with the expected complement of autosomes and X chromosomes were analyzed further.

Supplementary Figure 2

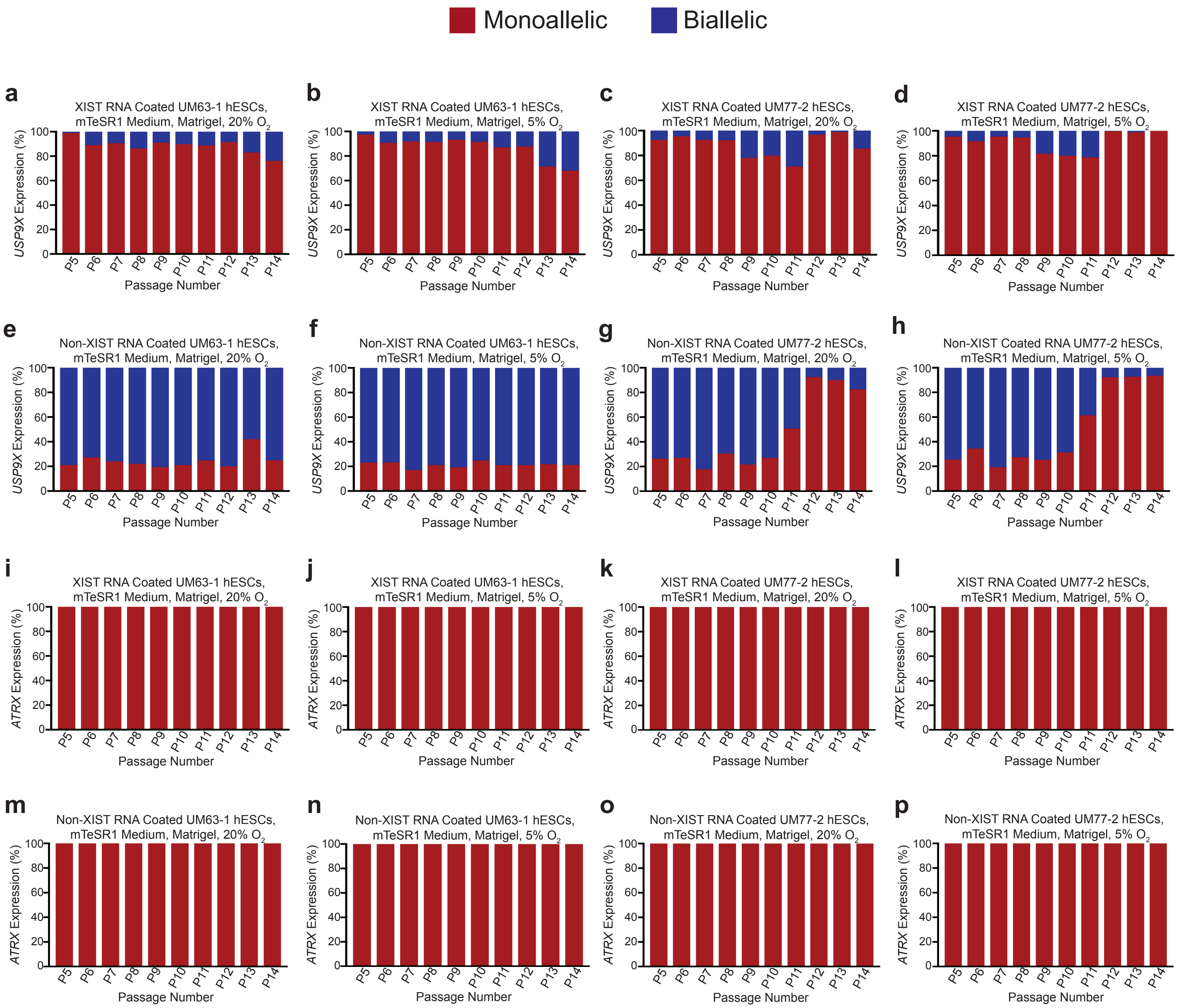

Supplementary Fig. 2. Impact of Atmospheric (20%) and Physiological (5%) O_2 Concentration on Expression of X-linked Genes *USP9X* and *ATRX* in female hESCs. (a-b) Expression pattern of nascent *USP9X* RNA in nuclei with XIST RNA coating in UM63-1 hESCs cultured under 20% **(a)** or 5% **(b)** O_2 detected by RNA FISH. **(c-d)** As in **a-b**, but with UM77-2 hESCs. **(e-f)** Expression pattern of nascent *USP9X* RNA in nuclei without XIST RNA coating in UM63-1 hESCs cultured under 20% **(e)** or 5% **(f)** O_2. **(g-h)** As in **e-f**, but with UM77-2 hESCs. **(i-j)** Expression pattern of nascent *ATRX* RNA in nuclei with XIST RNA coating in UM63-1 hESCs, cultured under 20% **(i)** or 5% **(j)** O_2. **(k-l)** As in **i-j**, but with UM77-2 hESCs. **(m-n)** Expression pattern of nascent *ATRX* RNA in nuclei without XIST RNA coating in UM63-1 hESCs cultured under 20% **(m)** or 5% **(n)** O_2. **(o-p)** As in **m-n**, but with UM77-2 hESCs. See also Figure 2. Source data are provided as a Source Data file.

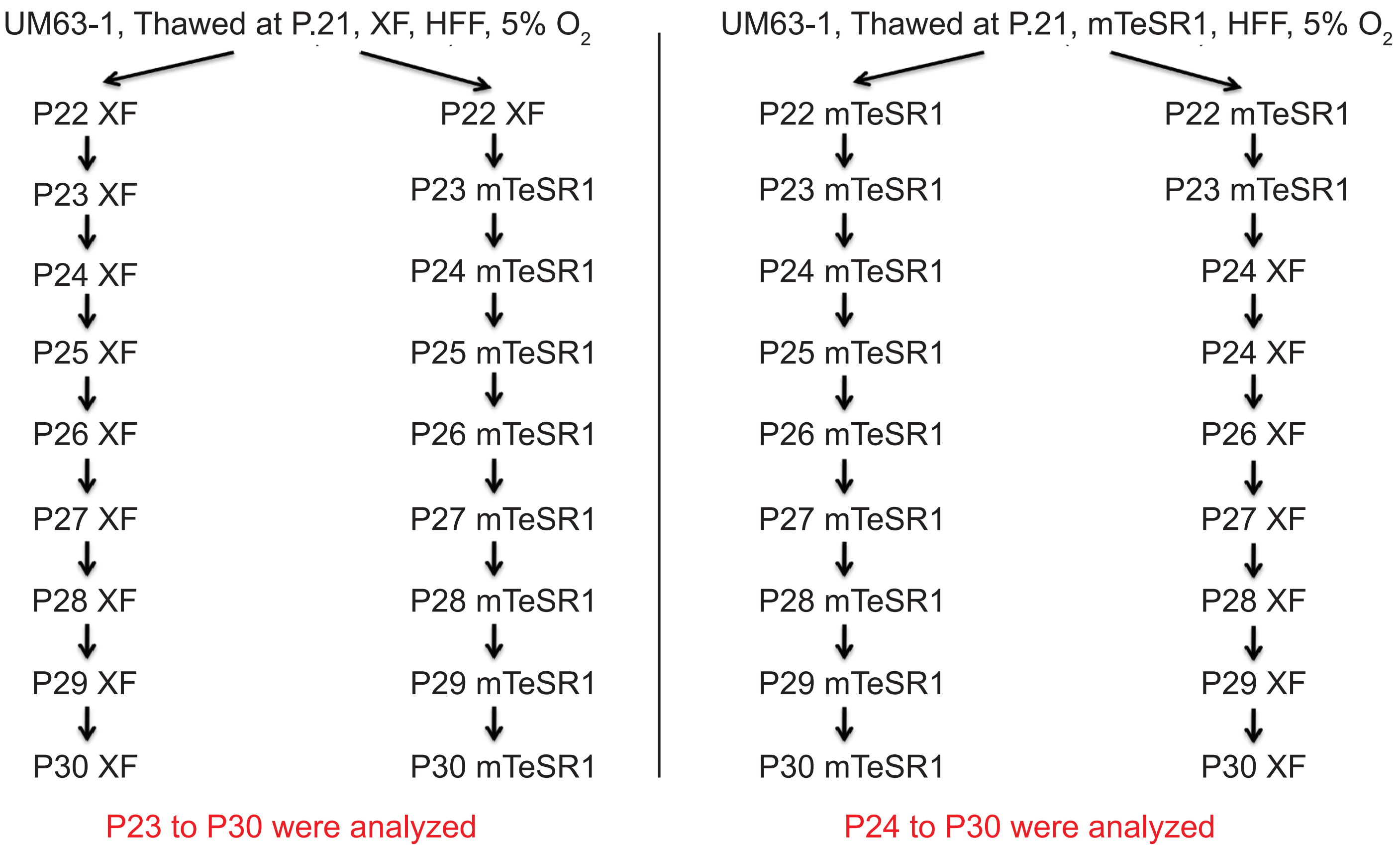
UM63-1, Thawed at P.21, XF, HFF, 5% O2
P22 XF
P23 XF
P24 XF
P25 XF
P26 XF
P27 XF
P28 XF
P29 XF
P30 XF
P22 XF
P23 mTeSR1
P24 mTeSR1
P25 mTeSR1
P26 mTeSR1
P27 mTeSR1
P28 mTeSR1
P29 mTeSR1
P30 mTeSR1
P23 to P30 were analyzed
UM63-1, Thawed at P.21, mTeSR1, HFF, 5% O2
P22 mTeSR1
P23 mTeSR1
P24 mTeSR1
P25 mTeSR1
P26 mTeSR1
P27 mTeSR1
P28 mTeSR1
P29 mTeSR1
P30 mTeSR1
P22 mTeSR1
P23 mTeSR1
P24 XF
P24 XF
P26 XF
P27 XF
P28 XF
P29 XF
P30 XF
P24 to P30 were analyzed

Supplementary Fig. 3. Strategy for Culture Media Switch Experiment in Figure 5.

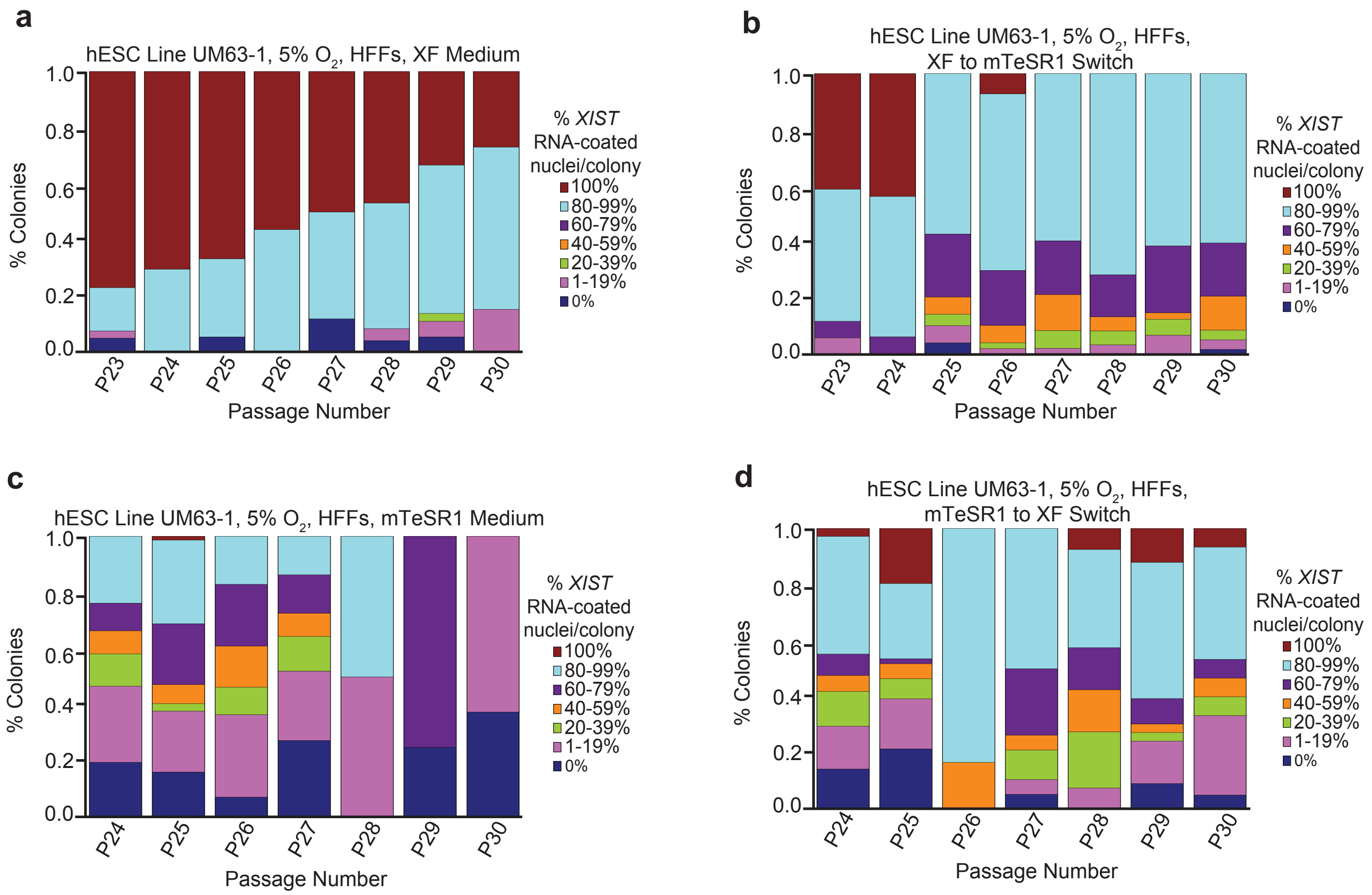
a
hESC Line UM63-1, 5% O2, HFFs, XF Medium
% Colonies
Passage Number
P23
P24
P25
P26
P27
P28
P29
P30
% XIST RNA-coated nuclei/colony
100%
80-99%
60-79%
40-59%
20-39%
1-19%
0%
b
hESC Line UM63-1, 5% O2, HFFs, XF to mTeSR1 Switch
c
hESC Line UM63-1, 5% O2, HFFs, mTeSR1 Medium
d
hESC Line UM63-1, 5% O2, HFFs, mTeSR1 to XF Switch

Supplementary Fig. 4. Detailed Analysis of Culture Media Switching on XIST RNA Coating. Stratification of XIST RNA FISH data from Figure 5 into seven categories of percentage of nuclei with XIST RNA coats per colony of hESC line UM63-1 **(a)** cultured continuously in XF medium and **(b)** cultured initially in XF medium and subsequently switched to mTeSR1 medium. Percentage of nuclei with XIST RNA coats per colony of hESC line UM63-1 **(c)** continuously cultured in mTeSR1 medium and **(d)** cultured initially in mTeSR1 medium and then switched to XF medium. hESCs cultured initially in XF medium and subsequently switched to mTeSR1 medium displayed a significant decrease in the proportion of nuclei with XIST RNA coating per colony during passaging compared to hESCs cultured continuously in XF medium (general linear model comparison, $p = 0.01$). hESCs cultured continuously in mTeSR1 medium displayed a significant decrease in nuclei with XIST RNA coating during passaging compared to those cultured initially in mTeSR1 medium and then switched to XF medium (general linear model comparison, $p < 0.001$). See also Figure 5 and Supplementary Fig. 4. Source data are provided as a Source Data file.

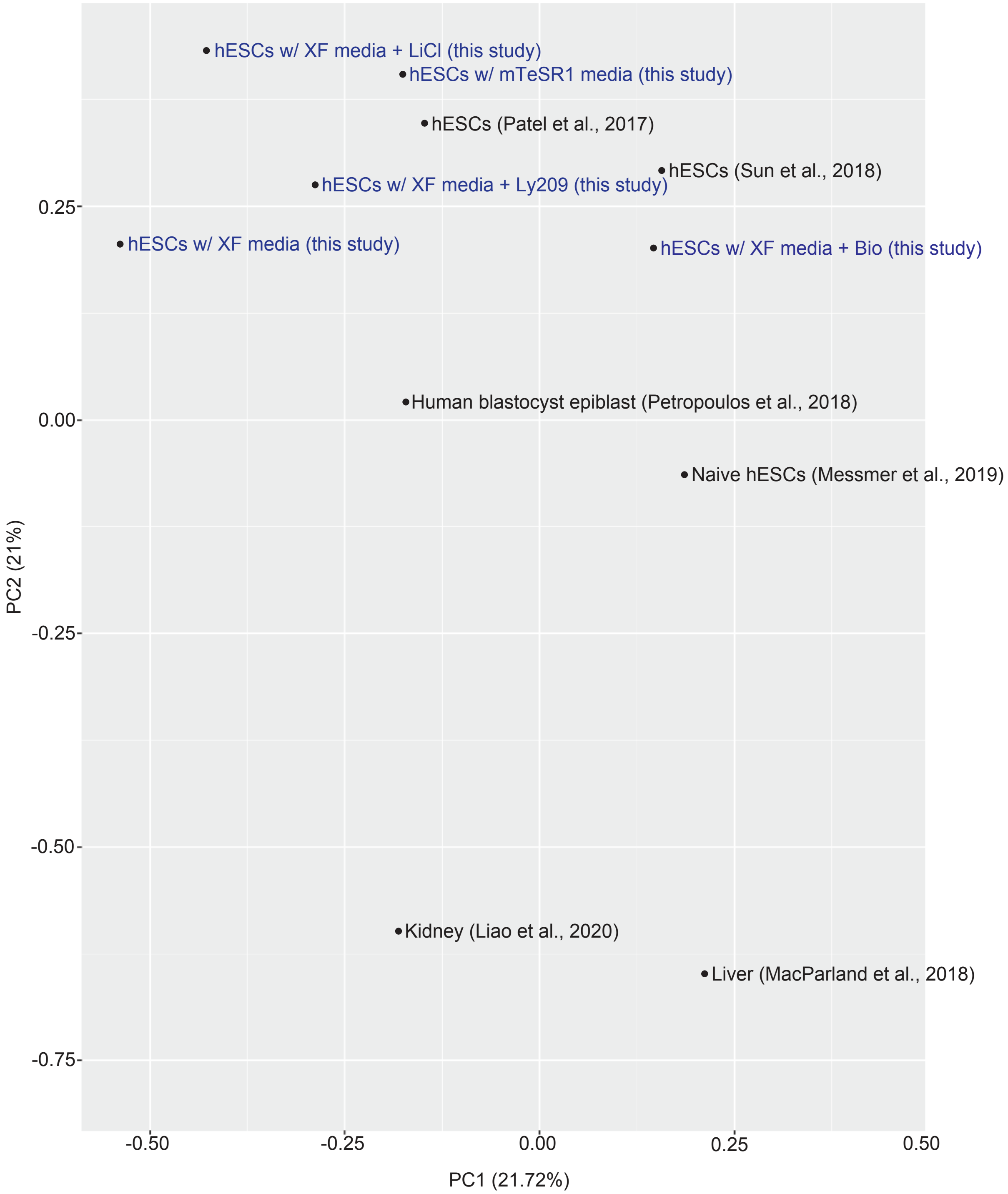
hESCs w/ XF media + LiCl (this study)
hESCs w/ mTeSR1 media (this study)
hESCs (Patel et al., 2017)
hESCs (Sun et al., 2018)
hESCs w/ XF media + Ly209 (this study)
hESCs w/ XF media (this study)
hESCs w/ XF media + Bio (this study)
Human blastocyst epiblast (Petropoulos et al., 2018)
Naive hESCs (Messmer et al., 2019)
Kidney (Liao et al., 2020)
Liver (MacParland et al., 2018)
0.25
0.00
-0.25
-0.50
-0.75
PC2 (21%)
-0.50
-0.25
0.00
0.25
0.50
PC1 (21.72%)

Supplementary Fig. 5. Transcriptome Comparison of hESCs, Human Blastocyst Epiblast, and Differentiated Cell Types. Principal component analysis of RNA-Seq data generated from P28 hESC line UM90-14 cultured using the following media formulations: XF medium; mTeSR1 medium; XF medium with 0.98mM LiCl; XF medium with 1.5 nM Ly2090314; XF medium with 5.0 nM BIO. Data generated for this study was compared to published RNA-Seq datasets[20, 46, 51, 98].

Source Data. **Raw RNA FISH Quantification Data for Figures 1-10 and Supplementary Figures 2 and 4 and differential expression analysis data.**
