# Supplementary material for: Preventing erosion of X-chromosome inactivation in human embryonic stem cells
Source: Nat Commun. 2022 May 6;13:2516. doi: 10.1038/s41467-022-30259-x (PMC9076865; doi:10.1038/s41467-022-30259-x)
Supplement: Supplementary file 3 — Reporting Summary [file 41467_2022_30259_MOESM3_ESM.pdf]

Corresponding author(s): Sundeep Kalantry

Last updated by author(s): December 2, 2021

Reporting Summary

Nature Research wishes to improve the reproducibility of the work that we publish. This form provides structure for consistency and transparency in reporting. For further information on Nature Research policies, see our Editorial Policies and the Editorial Policy Checklist.

Statistics

For all statistical analyses, confirm that the following items are present in the figure legend, table legend, main text, or Methods section.

n/a	Confirmed	
☐	☒	The exact sample size (*n*) for each experimental group/condition, given as a discrete number and unit of measurement
☐	☒	A statement on whether measurements were taken from distinct samples or whether the same sample was measured repeatedly
☐	☒	The statistical test(s) used AND whether they are one- or two-sided<br>*Only common tests should be described solely by name; describe more complex techniques in the Methods section.*
☐	☒	A description of all covariates tested
☒	☐	A description of any assumptions or corrections, such as tests of normality and adjustment for multiple comparisons
☐	☒	A full description of the statistical parameters including central tendency (e.g. means) or other basic estimates (e.g. regression coefficient) AND variation (e.g. standard deviation) or associated estimates of uncertainty (e.g. confidence intervals)
☐	☒	For null hypothesis testing, the test statistic (e.g. *F*, *t*, *r*) with confidence intervals, effect sizes, degrees of freedom and *P* value noted<br>*Give P values as exact values whenever suitable.*
☒	☐	For Bayesian analysis, information on the choice of priors and Markov chain Monte Carlo settings
☒	☐	For hierarchical and complex designs, identification of the appropriate level for tests and full reporting of outcomes
☒	☐	Estimates of effect sizes (e.g. Cohen's *d*, Pearson's *r*), indicating how they were calculated

Our web collection on statistics for biologists contains articles on many of the points above.

Software and code

Policy information about availability of computer code

Data collection	Expression of X-linked genes detected by RNA fluorescence in situ hybridization (FISH) was quantified at 10X to 100X resolution on a Nikon Eclipse TiE inverted microscope with a Photometrics CCD camera, starting at the upper left corner of each slide.
Data analysis	The RNA FISH data were statistically analyzed using a general linear model regression analysis (Figures 1-10). For figures comparing different culture conditions (Figures 2-6), p-values comparing the models generated for each condition were calculated. A threshold of p = 0.05 was used to test for statistical significance. All RNA FISH images were deconvolved and uniformly processed using NIS-Elements software (Version 4.60.00). For the RNA-Seq analysis, quality control was conducted using FastQC (Version 0.11.9). Reads were aligned to the hg19 (human) reference genome using STAR (Version 2.7.10a) and counted using FeatureCounts (Version 1.22.2). Differential expression analysis was conducted using DESeq2 (Version 1.34.0).

For manuscripts utilizing custom algorithms or software that are central to the research but not yet described in published literature, software must be made available to editors and reviewers. We strongly encourage code deposition in a community repository (e.g. GitHub). See the Nature Research guidelines for submitting code & software for further information.

Data

Policy information about availability of data

All manuscripts must include a data availability statement. This statement should provide the following information, where applicable:

- Accession codes, unique identifiers, or web links for publicly available datasets
- A list of figures that have associated raw data
- A description of any restrictions on data availability

All human embryonic stem cell (hESC) lines generated for this study have been deposited to the NIH Human Embryonic Stem Cell Registry (https://grants.nih.gov/stem_cells/registry/current.htm). All mouse ESC lines and EpiSC lines used in the study are available from the authors. All RNA FISH stain quantification data generated for this study can be found in the Source Data file. All RNA-Seq data have been deposited into the Gene Expression Omnibus (GSE157809).

Field-specific reporting

Please select the one below that is the best fit for your research. If you are not sure, read the appropriate sections before making your selection.

☒ Life sciences ☐ Behavioural & social sciences ☐ Ecological, evolutionary & environmental sciences

For a reference copy of the document with all sections, see nature.com/documents/nr-reporting-summary-flat.pdf

Life sciences study design

All studies must disclose on these points even when the disclosure is negative.

Sample size	Sample size calculations were not performed for RNA FISH quantification because all quantifiable (i.e. nonoverlapping) nuclei in each colony with more than 100 nuclei on each coverslip were quantified.
Data exclusions	No data were excluded from the analyses.
Replication	Key experiments (figures 2-4, 7, 8, 9) in this study were successfully replicated in two or more independent cell lines.
Randomization	Randomization is not relevant to our study because the same cell line and passage number was used for all treatments in each experiment.
Blinding	Investigators were not blinded, but this was not necessary as data collected were based solely on counting RNA FISH signals.

Reporting for specific materials, systems and methods

We require information from authors about some types of materials, experimental systems and methods used in many studies. Here, indicate whether each material, system or method listed is relevant to your study. If you are not sure if a list item applies to your research, read the appropriate section before selecting a response.

Materials & experimental systems

n/a	Involved in the study	
☐	☒	Antibodies
☐	☒	Eukaryotic cell lines
☒	☐	Palaeontology and archaeology
☒	☐	Animals and other organisms
E	☒	Human research participants
☒	☐	Clinical data
☒	☐	Dual use research of concern

Methods

n/a	Involved in the study	
☒	☐	ChIP-seq
☒	☐	Flow cytometry
☒	☐	MRI-based neuroimaging

Antibodies

Antibodies used	Mouse anti-TRA 1-60, pluripotency marker, 1:200 dilution, Millipore #MAB4360; Goat anti-OCT3/4, pluripotency marker, 1:300 dilution, Santa Cruz Biotechnology #sc-8628; Rabbit anti-SOX2, pluripotency marker, 1:800 dilution, EMD Millipore #AB5603; Rabbit anti-NANOG, pluripotency marker, 1:150 dilution, Abcam #ab21624; Mouse anti-SSEA4, pluripotency marker, 1:100 dilution, Millipore #MAB4304; Donkey anti-Rabbit, secondary antibody, NANOG 1:200 dilution, SOX2 1:800 dilution, Jackson ImmunoResearch #711-165-152; Donkey anti-Goat, secondary antibody; 1:800 dilution, Jackson ImmunoResearch #705-096-147; Donkey anti-Mouse, secondary antibody; TRA 1-60: 1:100 dilution; SSEA4: 1:800 dilution, Jackson ImmunoResearch #715-165-150.
Validation	Per the Millipore website, the mouse Anti-TRA-1-60 antibody (#MAB4360), clone TRA-1-60 is validated for use in WB, FC, IF, IP, IC for the detection of TRA-1-60. Per the manufacturer's website, the goat anti-OCT3/4 antibody (#sc-8628) has been used in 76 published studies, including Tahmasebi et al., 2016, Respuela et al., 2016, and Abboud et al., 2015. Per the Millipore website, the Anti-SOX2 Antibody, Cat. No. AB5603, is a highly specific rabbit polyclonal antibody SOX2 and has been tested for use in Immunocytochemistry, and Immunohistochemistry (Paraffin), and Western Blotting. Per the Abcam website, the rabbit anti-NANOG antibody (#ab21624) has been cited in 169 publications and validated for use in ICC, ICC/IF, WB, and Sandwich ELISA. According to the Millipore website, Anti-Stage-Specific Embryonic Antigen-4 Antibody (#MAB4304), clone MC-813-70 is an antibody against Stage-Specific Embryonic Antigen-4 for use in FC, ELISA, IF, IH.

Eukaryotic cell lines

Policy information about cell lines

Cell line source(s)	All hESC lines used in the study were derived from human embryos generated for infertility treatments and were donated to the University of Michigan for one of two reasons: 1) they were no longer needed for reproductive purposes by the donating couple; or 2) they were considered to be unsuitable for implantation following preimplantation genetic testing.

Authentication	We checked for the expression of pluripotency markers in each hESC line and also tested for the ability of each hESC line to differentiate into the three germ layers of the gastrulating embryo. Each hESC line was also subjected to embryoid body differentiation to determine the ability of the hESC line to generate mesoderm, endoderm, and ectoderm. We also determined that each hESC line has a normal female karyotype with two X-chromosomes using array CGH and/or metaphase spreads. Each hESC line was also subjected to DNA fingerprinting via short tandem repeat analysis to test for the uniqueness of each hESC line.
Mycoplasma contamination	Mycoplasma contamination of all hESC lines used in the study was tested for using a PCR-based assay (Sigma, MP0025) and gel electrophoresis. The assay contained a PCR-negative control (no polymerase), a positive control (non-infectious DNA fragments of Mycoplasma orale genome, band size 267bp), and an internal control (internal sequence of HTLV-I tax gene, presence of amplification band at 191bp and no band at 267bp). All lines tested negative for mycoplasma.
Commonly misidentified lines (See ICLAC register)	No cell lines used in this study are commonly misidentified.
